# Supplementary material for: Description and Whole-Genome Sequencing of Mariniflexile litorale sp. nov., Isolated from the Shallow Sediments of the Sea of Japan
Source: Microorganisms. 2024 Jul 12;12(7):1413. doi: 10.3390/microorganisms12071413 (PMC11278836; doi:10.3390/microorganisms12071413)
Supplement: Supplementary file 1 [file microorganisms-12-01413-s001.zip › Certificate of deposit _KMM 9835.pdf]

G.B. Elyakov Pacific Institute of Bioorganic Chemistry
Far Eastern Branch, Russian Academy of Sciences

159 100-let Vladivostoku Prospect, Vladivostok, 690022, Russia. ☎ 7(423) 231-14-30; fax: 7(423) 231-40-50

Collection of Marine Microorganisms

CERTIFICATE of DEPOSIT and AVAILIBILITY

This is to certify that the type strain of "*Mariniflexile litorale*" sp. nov. KMM 9835^T has been deposited and is maintained in the Collection of Marine Microorganisms (KMM), G.B. Elyakov Pacific Institute of Bioorganic Chemistry, Far-Eastern Branch, Russian Academy of Sciences. The type strain of "*Mariniflexile litorale*" sp. nov. KMM 9835^T will be available to the public after publication of the manuscript that describes this species.

Signature___________

Name: Mikhailov V.V., Ph.D., D. Sc.
Professor of Microbiology
Curator of Collection of Marine Microorganisms
G.B. Elyakov Pacific Institute of Bioorganic Chemistry,
Far-Eastern Branch, Russian Academy of Sciences,
Prospect 100-let Vladivostoku, 159,
690022 Vladivostok,
Russia

Place and date: Vladivostok, December 7, 2021
