# Supplementary material for: Description and Whole-Genome Sequencing of Mariniflexile litorale sp. nov., Isolated from the Shallow Sediments of the Sea of Japan
Source: Microorganisms. 2024 Jul 12;12(7):1413. doi: 10.3390/microorganisms12071413 (PMC11278836; doi:10.3390/microorganisms12071413)
Supplement: Supplementary file 1 [file microorganisms-12-01413-s001.zip › KCTC 92792.pdf]

**Korean Collection for Type Cultures (KCTC)**
Korea Research Institute of Bioscience and Biotechnology (KRIBB)
181 Ipsin-gil, Jeongeup-si, Jeonbuk 56212, South Korea
Tel: +82-63-570-5602, FAX: +82-63-570-5609
E-mail: deposit@kribb.re.kr

# Certificate of Deposit

Ref.: 20230125 Date of issue: FEB 8, 2023

| | | |
|---|---|---|
| Taxonomic designation | : | ***Mariniflexile* sp.** |
| Accession number | : | **KCTC 92792** |
| Depositor(s) | : | Valeriya Kurilenko |
| Strain code by the depositor(s) | : | **KMM 9835** |

The above microorganism has been successfully deposited into the general collection of microorganism of the Korean Collection for Type Cultures (KCTC) and confirmed the identity of the microorganism under this KCTC number.
This microorganism will be available without restrictions for research and academic purposes in the publicly accessible section of the KCTC. It will be included in published and online catalogues after publication of this number by the authors.

Curator of Bacteria & Archaea
Na-Ri Shin, Ph.D.

Telephone: +82-63-570-5616
Fax: +82-63-570-5609
E-mail: nrshin@kribb.re.kr
Web: http://kctc.kribb.re.kr
