# Supplementary material for: Description and Whole-Genome Sequencing of Mariniflexile litorale sp. nov., Isolated from the Shallow Sediments of the Sea of Japan
Source: Microorganisms. 2024 Jul 12;12(7):1413. doi: 10.3390/microorganisms12071413 (PMC11278836; doi:10.3390/microorganisms12071413)

Description and Whole-Genome Sequencing of *Mariniflexile litorale* sp. nov., Isolated from the Shallow Sediments of the Sea of Japan

Lyudmila Romanenko [1,*], Evgeniya Bystritskaya [1,†], Yuliya Savicheva [1,†], Viacheslav Eremeev [1], Nadezhda Otstavnykh [1], Valeriya Kurilenko [1], Peter Velansky [2] and Marina Isaeva [1,*]

[1] G.B. Elyakov Pacific Institute of Bioorganic Chemistry, Far Eastern Branch, Russian Academy of Sciences, Prospect 100 Let Vladivostoku, 159, Vladivostok 690022, Russia; ep.bystritskaya@yandex.ru (E.B.); iu.savicheva0@yandex.ru (Y.S.); wieremeew@gmail.com (V.E.); chernysheva.nadezhda@gmail.com (N.O.); valerie@piboc.dvo.ru (V.K.)

[2] A.V. Zhirmunsky National Scientific Center of Marine Biology, Far Eastern Branch, Russian Academy of Sciences, Palchevskogo Street 17, Vladivostok 690041, Russia; velansky.pv@gmail.com

***** Correspondence: lro@piboc.dvo.ru (L.R.); issaeva@gmail.com (M.I.); Tel.: +7-423-231-1168 (L.R.)

† These authors contributed equally to this work.

Supplementary materials

Supplementary Figure S1.

NJ/ML/MP tree based on 16S rRNA gene sequences available from the GenBank database showing relationships of the novel strain KMM 9835^{T} (in bold), *Mariniflexile* species and related taxa of the family *Flavobacteraceae*. NJ tree was reconstructed using the Kimura two–parameter model and ML tree was inferred under the GTR+GAMMA model. The branches are scaled in terms of the expected number of substitutions per site. The numbers above the branches represent bootstrap values with 1000 replicates larger than 60% (NJ/ ML/MP). The bar indicates 0.02 accumulated substitutions per nucleotide position.

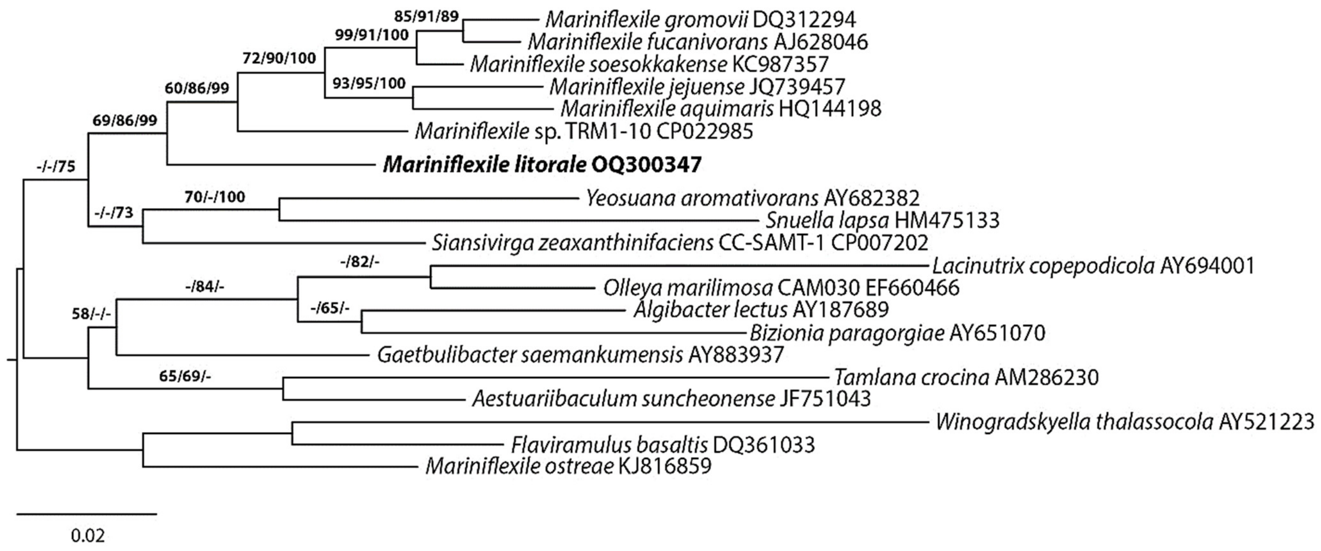

Supplementary Figure S2.

ML tree based on concatenated sequences of 400 proteins showing phylogenetic position of strain KMM 9835^T, AS56 and TRM1-10 among *Mariniflexile* species and related taxa. Bootstrap values are based on 100 replicates. Bar, 0.20 substitutions per amino acid position.

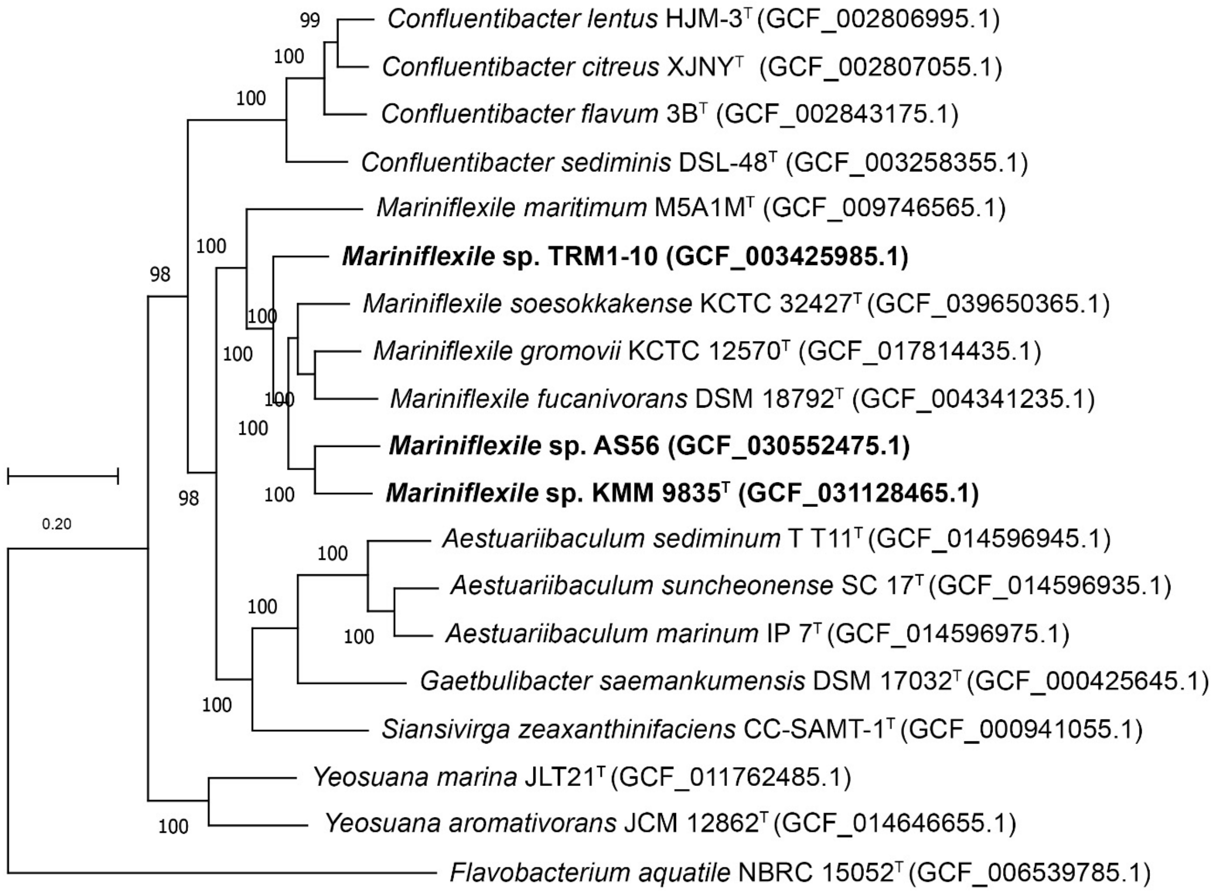

Supplementary Figure S3.

Pan-genome modelling. (a) Gene accumulation curves for the pan-genome (blue curve) and the core (green curve) genome of seven *Mariniflexile* genomes. Pan-genome curve: $y = 2634.4x^{0.58} + 925.34$. Core genome curve: $y = 6139.3e^{-1.24x} - 1780.27$. (b) The new gene cluster number plot, curve: $y = 1835.82x^{-0.49}$. Pan-genome openness was estimated under Heap's law model [54].

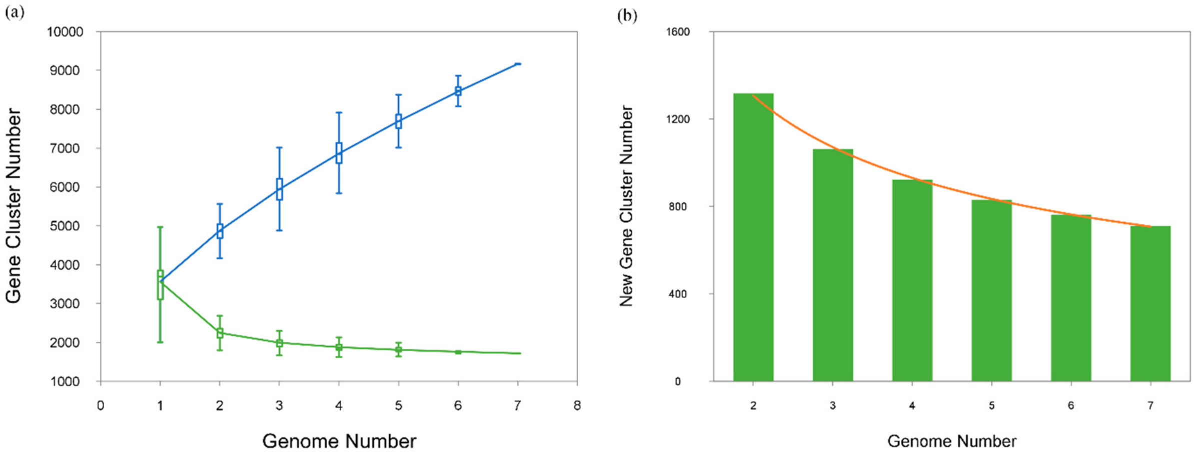

Supplementary Figure S4.

Two-dimensional thin-layer chromatograms of polar lipids of strains: (a, b, c) KMM 9835^T; (d, e, f) *Mariniflexile maritimum* KCTC 72895^T; (g, h, i) *Mariniflexile soesokkakense* KCTC 32427^T. Data were obtained from present study. (a, d, g), non-specific detection of lipids prepared with 10% H_2SO_4 in methanol; (b, e, h), stained with ninhydrin; (c, f, i), stained with molybdate reagent. Abbreviations: PE, phosphatidylethanolamine; AL1, AL2, unidentified aminolipids; PL, an unidentified phospholipid; AL, an unidentified aminolipid; L1-L6, unidentified lipids.

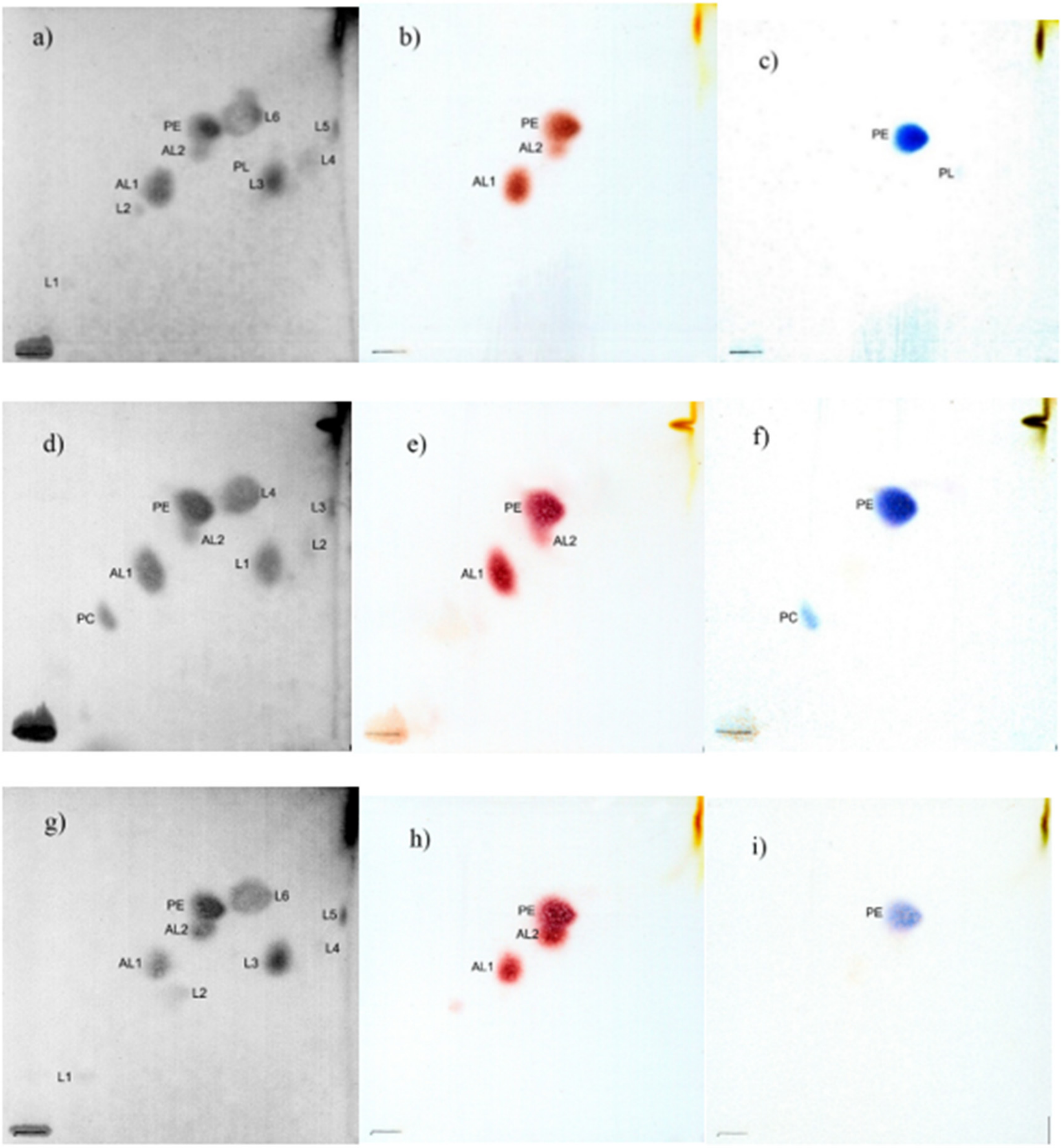

Supplement: Supplementary file 1 [file microorganisms-12-01413-s001.zip › Supplemenary.pdf]
